# Will the Artificial Intelligence Touch Substitute for the Human Touch?

**DOI:** 10.3390/neurosci5030020

**Published:** 2024-07-24

**Authors:** Laura Clara Grandi, Stefania Bruni

**Affiliations:** 1 Department of Biotechnology and Biosciences, NeuroMI (Milan Center of Neuroscience), University of Milano-Bicocca, Piazza della Scienza 2, 20126 Milano, Italy; 2 Centro Cardinal Ferrari, Fontanellato, Via IV novembre 21, 43012 Fontanellato, Italy; stefania.bruni@centrocardinalferrari.it

**Keywords:** artificial intelligence, social touch, BioTac^®^, robotic arms, robotic hands, touch, affective touch

## Abstract

Nowadays, artificial intelligence is used in many fields to diagnose and treat different diseases. Robots are also useful tools that substitute for human work. Despite robots being used also for touch therapy, can they substitute for the human touch? Human touch has a strong social component, and it is necessary for the correct development of newborns and the treatment of pathological situations. To substitute human touch, it is necessary to integrate robots with artificial intelligence as well as with sensors that mimic human skin. Today, the question remains without answer: Can human touch be substituted with AI in its social and affiliative components?

## 1. Introduction

Social touch can be defined as intentional physical contact between individuals. Its role is crucial in human development [1] and psychological well-being [2] as well as in the creation and maintenance of social interactions [3,4]. The significance of social touch is further emphasized by the evidence indicating its encoding within a distinct brain network, known as the *social brain*, which differs from the circuit responsible for encoding the discriminative aspect of touch. Within this circuit, the insular cortex plays a pivotal role [5].

People who live alone are deprived of social touch and experience high levels of stress as well as symptoms of mood and anxiety disorders [6]. Similarly, the recent pandemic [7], which has obligated governments to enforce isolation by quarantine and restriction rules to avoid the spread of COVID-19, determined the absence of interpersonal touch.

The era of artificial intelligence (AI) seems to be replacing humans in many fields despite the importance of the human factor in social touch. Recently, Eckstein and colleagues [8] highlighted the effect of touch between humans and artificial objects, but will AI be able to replace the human touch? Will this substitution be necessary?

How is AI involved in the touch field? In some studies related to touch perception, tactile stimulation is performed by a machine. Interestingly, in healthy participants, tactile stimulation is evaluated as equally pleasant as when delivered manually by humans [9]. Moreover, Feng and colleagues [10] proposed the use of AI for Chinese massage therapy. Despite the high precision of massage techniques, it is still necessary to improve the efficacy and safety of massage equipment or robots [11,12].

Artificial intelligence also finds a significant application in the field of prosthetic devices, especially when the utilization of robotic hands is involved. It is crucial to create prosthetic devices that enable users to interact with objects through precise movements while also simulating the sensation raised by touch. This goal has been recently achieved [13]. In particular, the BioTac^®^ is a tactile sensor able to detect point of contact as well as object spatial properties with impedance sensing electrodes [14], micro-vibrations, and textures, thanks to a hydro-acoustic pressure sensor and thermistor, respectively [15,16,17].

Given that (1) robotic arms are currently utilized in experiments investigating social touch perception and (2) the robotic fingers of prostheses possess advanced properties enabling tactile sensation, the question of whether human touch may soon be entirely replaced by machines arises. This narrative review aims to (1) summarize the primary characteristics and applications of social touch across various physiological and pathological conditions, (3) underline the importance of social touch, (4) explore the potential role of AI and of robots in these domains, (5) raise ethical concerns of AI and robot use, and (6) address the following question: “Can machines replicate human touch?”. We believe this review will serve as a focal point for contemplating the possibilities offered by emerging technologies that supplement the human touch together with the limitations and the boundaries to consider for the ethical and conscious use of digital technologies.

## 2. Materials and Methods

A literature search was conducted on PubMed and Embase from January to June 2024 using the following terms: “social touch” and/or “artificial intelligence” and/or “robotic arm” and/or “robotic hand” in the title and/or abstract and/or “BioTac” in the method section, with no restrictions for the publication year and type of publications. Google searches were conducted on applications of robots and robots integrated with AI.

We used the Grammarly tool (https://www.grammarly.com/ accessed on 6 May 2024) just to correct and rephrase some sentences with professional English. Grammarly was not used to write this review.

## 3. Results

**Figure 1:**
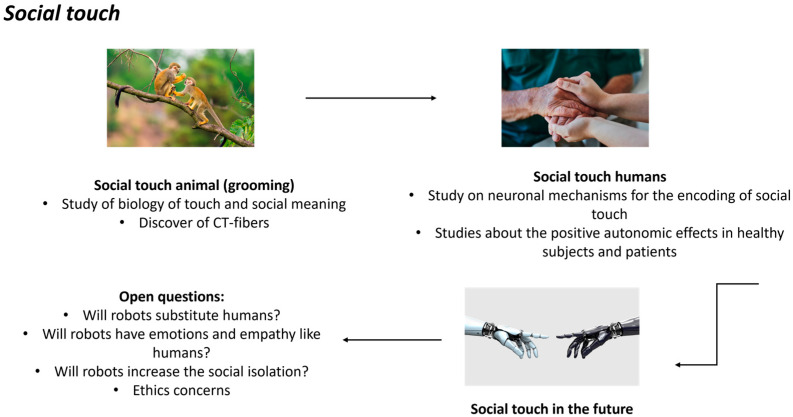
The social touch in animals and in humans in the future.

We will present a resume of literature about the social touch and its importance, from the experiments of Harlow and Zimmerman in rhesus monkeys to human society today, for the treatment of different diseases and then explore the use of AI in the future of social touch. Figure 1 resumes the social touch in animals (grooming) and in humans (caress-like; see [18] for a review about the possible evolution of touch from non-human to human primates) toward the future of touch with the use of AI.

### 3.1. Social Touch

Unlike discriminative touch that allows for an individual to discriminate objects and recognize their features, e.g., shape and form, social touch is a tactile stimulation characterized by a specific speed and pressure [19,20,21]. Because of the positive autonomic effects, social touch has been used in different pathological contexts, e.g., in preterm newborns to induce immune system development, in neonatal intensive care [22,23], in parent–child interventions [24], in the oncological clinical field to induce relaxation [25,26], in mood disorders such as depression [27], and in multiple sclerosis patients to improve their quality of life [28]. Interestingly, it is also used in behavioral therapies for autism spectrum disorders. Even if autistic patients tend to reject physical contact, remarkably, this touch can positively modulate their behaviors [29].

Social touch is mediated by distinct skin receptors [30], and it is linked to positive physiological outcomes, such as decreased heart rate and increased heart rate variability [27,31]. It is also processed by dedicated neural circuits in the brain. After the activation of the C-tactile (CT) fibers on the hairy skin [30,32,33,34,35], the signal is encoded by the central “social brain” network [20,21,35,36].

This tactile stimulation affects blood pressure and cortisol levels. It also triggers oxytocin release, which is a hormone that promotes emotional bonding. Moreover, touch is positively associated with immune system functions [21,37]. Globally, the association between touch and positive feelings has been demonstrated [38]. These properties also highlight the importance of tactile stimulation as a modality of social communication.

### 3.2. The Importance of Social Touch

In 1958, Harlow and Zimmerman showed the importance of touch. In a well-known experiment involving baby macaque monkeys, the monkeys had to choose between two surrogate mothers made of wire mesh. One provided food, while the other was made of terrycloth but did not provide food. Surprisingly, the monkeys showed a clear preference for the terrycloth mother. This experiment highlighted that touch was considered more important to the monkey infants than nourishment [39,40]. The importance of touch in non-human primates is widely demonstrated, and recently, the importance of allogrooming in adult monkeys has been reported by observing monkeys housed in adjacent enclosures separated by a glass panel. In that study, the authors reported that the monkeys imitate grooming and that this virtual grooming had similar physiological sensations and social effects as real allogrooming. This observation is important because it underlines the importance of social touch to maintain social bonds, even in a situation where physical direct contact is not possible [41].

The importance arises during pregnancy. Indeed, in humans, touch is the first sense to emerge during ontogenesis at approximately eight weeks of gestation [42]. Moreover, the fetus responds to vibration (vibroacoustic stimulation) from 29 weeks of gestation with the acceleration of the heart rate [43], and this response stabilizes by 32 weeks of gestation [44]. The fetus constantly touches the environment as well as its body, preferring the body areas that are densely innervated, e.g., the skin of the face that is rich in trigeminal innervation. Other body areas, such as the stomach, thorax, or back, are rarely touched, but they are often passively touched or pushed against the uterine wall [44]. In 2015, Marx and Nagy reported the fetal responsiveness to maternal voice and touch and found an increase in fetal body movements when the mother touched the abdomen. Moreover, the fetuses in the third trimester touched the uterine wall significantly longer than in the second trimester when the mother touched it. This difference can be explained by the maturation of the central nervous system, which leads to the emergence of proprioceptive self-awareness by the third trimester [45].

Tactile perception is also important for and during the development of the mature organism [46]. Evidence of the importance of touch in newborns came from studies with children living in a situation of touch deprivation. For example, Carlos and Earls [47] reported that touch-deprived children in understaffed orphanages in Romania had lower cortisol and growth development levels for their age group. Moreover, premature neonates show facilitated growth; increased weight gain [48]; and better cognitive development, orientation, and motor skills [49] if they are touched with a massage.

Skin-to-skin care is a type of tactile stimulation that is beneficial for both mothers and babies. Several empirical studies and clinical guidelines have supported its use, and it has become common in hospitals worldwide. This type of care is effective in reducing stress levels in mothers [50] and provides various benefits for infants [49]. Kangaroo mother care (KMC, i.e., skin-to-skin contact between mother and newborn) is recommended as the gold standard in all medical and nursing care [51]. It has been effective in reducing the risk of mortality among preterm and low-birth-weight infants. Moreover, its use has been suggested for neonatals in intensive care [52]. Given the importance of maternal contact during early life, several hospitals have launched volunteer cuddling programs for all infants in the neonatal intensive care unit. Volunteers are trained to cuddle infants when caregivers are not available. In Italy, there is the “donatori di coccole”, an association of volunteers educated by physicians and psychologists to give massages to neonates who cannot receive a touch from parents [53].

In the recent COVID-19 pandemic, many people experienced social deprivation, particularly those living alone who were deprived of touch with others, which negatively impacted their well-being [54,55]. Since the physical distancing regulations during the COVID-19 pandemic, our ability to provide and receive social touch has been affected. Previous works on touch deprivation showed a link between touch deprivation and altered perceived pleasantness of CT-optimal touch, e.g., in infants and children in institutional care [55]. Interestingly, Meijer and colleagues [7] showed that changes in touch frequency during the COVID-19 period might be associated with how CT-optimal touch is evaluated and how individuals can benefit in terms of mental health from this touch. In detail, participants in complete lockdown perceived the CT-optimal touch video to be significantly more pleasant than those who were under social distancing measures and those in lockdown. Moreover, it seems that longing for touch was not associated with the type and severity of the regulations, e.g., social distancing or complete lockdown, but with the duration of the regulations and the living conditions. Participants who lived alone or with pets reported being significantly more touch-deprived than participants who lived with housemates [7].

Finally, social touch is also used in adults living in different pathological situations to improve symptoms, for example, patients with borderline personality disorder, depression, mood disorders, autism, breast cancer, or multiple sclerosis [23,25,26,27,56]. Interestingly, specific massage in patients with Parkinson’s disease seems to improve motor functions [57] and alleviate chronic pain [58]. Recently, we reviewed the use of social touch in different physiological and pathological conditions [59].

### 3.3. Artificial Intelligence for Social Touch

The term artificial intelligence (AI) was coined by John McCarthy in 1956 during a conference, but in 1950, Alan Turing, who developed the Turing test to differentiate humans from machines, described AI as similar to but more complex than the human brain [60,61].

Nowadays, AI is applied in many medical fields from diagnosis to treatments, with a higher accuracy and a lower human workload. Moreover, it also plays a crucial role in medical drug production or medical education [62]; in many medical areas, such as oncology and cardiology [63]; and the discovery of specific biomarkers to allow for an exact diagnosis among similar neurodegenerative conditions or even early diagnosis [64,65]. Moreover, we can speak about AI Plus [64].

In 1943, McCulloch invented an artificial neuron with binary function, and today, it is available as an artificial neural network (ANN) inspired by the central nervous system and made of interconnected processors (neurons) that perform parallel computations for data processing. ANNs are used in medicine since they help solve clinical problems, depending on a complex interaction of clinical, biological, and pathological variables [66].

A subfield of AI is deep learning, which is, in turn, a subfield of machine learning. One of the uses of deep learning is the diagnosis of cancers based on the classification of radiological or pathological images [67].

### 3.4. Robots and Artificial Intelligence

Another use of AI is the creation of robots integrated with AI. Indeed, AI is more complex than robots because they are created to perform specific actions, whilst AI simulates the human mind. Although many robots today are built without AI because they only need to perform simple, repetitive tasks, integrating AI can be useful in some cases to create robots that emulate the human brain. Currently, robots integrated with AI are used in the healthcare, business, and aerospace fields. For instance, the “Waldo Surgeons” are robots able to perform medical-related operations with extreme precision and accuracy, allowing for clinicians to avoid being overstressed [68]. Starship Technologies developed delivery robots integrated with AI, maps, and sensors to package and deliver [69]. NASA and the space agencies of different countries are working on improving space exploration by developing robots with AI [70,71].

Can we have robots that mimic and substitute human touch? If robots have the human sense of touch, they will be able to interact with us even in different situations. The artificial skin must be similar to that of humans, i.e., soft, stretchable, robust, and capable of simultaneously detecting contact location with high resolution and intensity with high sensitivity. In this context, accurate tactile sensors and artificial skin are being developed [72].

In their research, Massari and colleagues [73] developed an artificial skin, recreating the slowly adapting type 2 Ruffini corpuscles by means of photonic transducers embedded in a soft polymeric matrix. External tactile stimuli are converted into photonic signals and decoded by an algorithm to deduce both the location and the magnitude. The artificial skin can be used to cover large areas, such as the limbs and bodies of robots, which allows for them to interact with humans.

The BioTac^®^ sensor is frequently utilized in various research fields due to its ability to replicate the sensory features of human hands and features like force, vibration, and temperature. Moreover, this sensor is used in prosthetic hands to improve the capacity of perception and touch [74,75,76]. This sensor allows for users to pick up objects (even fragile ones) without having visual feedback. Importantly, it presents an algorithm that mimics the inhibitory reflex, facilitating grasping without the users considering the amount of force used, similar to the reflexes of a real human hand [77].

Su and colleagues [17] used BioTac^®^ to develop an exploratory and perceptual algorithm for a mechatronic robotic system. The electrical resistance of the conductive liquid trapped between the elastomeric skin and a cluster of four electrodes on the flat fingertip surface of the rigid core of the BioTac^®^ allows for the measurement of the force and its distribution and, therefore, the control of exploratory motions. The lateral electrodes and the hydraulic pressure allow for the measurement of the skin deformation correlated with the material of touched objects.

AI is also used in Chinese medicine treatments, such as acupuncture, Tui Na massage, and Qigong practitioners. Nevertheless, the AI application is in the preliminary stage. For example, researchers are working to develop robots based on the Tui Na protocol to improve both its efficacy and safety (see 10 for a review in traditional Chinese medicine). Huang and colleagues [12] have developed a four-degree-of-freedom anthropomorphic robotic arm with complete elastic joints. This robotic arm is programmed with Chinese massage techniques, but it also allows for implementations according to the individual symptom. This aspect is important since it combines AI and traditional therapeutic methods. Despite intelligent systems for Tui Na massage allowing for many different functions, they are expensive; therefore, they are not widely used.

Wang and colleagues [11] introduced a portable back massage robot that can implement three different massage techniques, i.e., percussion, rolling, and kneading, on the back. The use of massage robots has been increasing rapidly due to their ability to provide higher precision in massage techniques, improving the coverage of massage areas and enhancing the massage effects. This allows for physicians to offer critical medical services while also ensuring the efficient allocation of medical resources [78]. However, there is still a need to improve the integration of sophisticated AI algorithms to allow for robots to treat different syndromes.

Among the devices developed to provide tactile contact, the “keep in touch” system allows for physical intimacy for physically distant people [79]. It is composed of a touch screen that presents a blurred image of each partner, and the image of a specific body part is brought into focus when it is touched by the partner. Nevertheless, there is no evidence about its effectiveness. Similarly, devices that mimic a hug have been created, e.g., one that mimics an actual hug with a vest that inflates [80]. A “hug shirt” has been created to allow for the detection of (1) the strength and the warmth of a hug and (2) the heart rate of the hugger. However, these devices have not been empirically tested [81].

Pet therapy is used for the treatment of dementia, and robotic pet therapy is seen as a viable substitute for animal therapy [82] where allergies, infections, biting, scratching, or fear of the animals are present and an obstacle for the therapy itself [83]. The PARO (short for “personal robot” in the Japanese language) robotic pet has been used in different countries since 2003 (FDA approved). It looks like a baby harp seal, is covered with an artificial fur, and has a hard inner skeleton. Under the skeleton, there is dual-processor control software that allows for both the generation of behavior and the recognition of voice. Paro is able to imitate animal behavior and to react to stimuli. Finally, it is useful to decrease stress and anxiety or even improve depression [84]. Additional positive effects of PARO are the improvement of oxygenation and cardiac status [82].

Until now, most of the work on touch with robots has focused on the calming effects of zoomorphic or pet-like robots in the realm of robot-assisted therapies (see [85] for an overview). Robots have a passive role, since the person caresses and touches them.

A type of robot used for human–robot interaction studies is a toy robot called Pekoppa [86], shaped like a bilobed plant, with leaves and a stem that make a nodding response as a reaction to sounds of speech [87].

The use of robots is helpful in the treatment of autism. Indeed, Kozima and colleagues [88] observed interactive behaviors with the robot Keepon. Importantly, the study by Giannopulu and Pradel [89] showed that a toy robot could be useful to treat the social-related skills of autistic children.

Moreover, the integration of robots with different sensory systems, e.g., visual and tactile, could be critical and useful. In this context, to increase the body contact of robot–humans, a soft plane tactile sensor has been developed that covers the robot itself. Thanks to this sensor, it is possible for the robot to detect both position and force of the touch [85].

### 3.5. Ethical Concerns of AI and Robots

The use of AI raises many questions about ethics. Recently, Elendu and colleagues [90] explored the ethical implications of AI and robotics in healthcare, underlining the ethical concerns that require attention and also the positive aspects of such integration, as a possibility of interdisciplinary collaboration. Importantly, AI and robotics foster multidisciplinary collaboration and the possibility of synergy between healthcare professionals and AI to optimize time-preserving human expertise [91]. Nevertheless, an ethical concern is the possible dehumanization of care. One case is the Paro robot that provides comfort in elderly care homes. Its use raises questions like the following: Can it completely substitute human interactions? Will it contribute to social isolation?

Similarly, robots cannot have emotions as we defined them: Could this damage the human–robot interactions? Will there be a risk of manipulation of human emotions? Moreover, one of the most delicate concerns is the safety of privacy and data protection, particularly true for AI use in healthcare where AI systems analyze and backup a vast amount of sensitive patient information.

In our opinion, one of the concerns about the use of robots and AI for social touch is the use of the definition of “social touch” itself. Does “Social” refer just to humans and animals but not to robots that have no ability to understand and decide, neither empathy nor fell emotions? In this context, the social robots are critical, i.e., robots able to interact with both humans and other robots (see [92] for a review on social robots). To do this, they have sensors, cameras, microphones, and other technology that allow for them to respond to touch, sounds, and visual cues similar to humans. One example is Furhatrobotics [93], which has the ability to participate in a conversation through natural human-like movements, e.g., headshaking and raising eyebrows. Another example is the Pepper robot, i.e., a humanoid robot created in 2014 able to exhibit body language and to interact and move around in its surroundings. Importantly, it can analyze people’s expressions and voice tones, and it is equipped with high-level interfaces for multimodal communication with humans [94].

Given this evidence, does the question “Will robots be as human in terms of empathy and emotions and feeling touch?” start to have an answer?

## 4. Discussion

Social touch plays a pivotal role in human development, psychological well-being, and the maintenance of social interactions. The neurological underpinnings of social touch involve distinct brain networks, particularly the insular cortex, which is specialized for processing social touch stimuli. The absence of social touch, as experienced during periods of isolation such as the recent COVID-19 pandemic, can lead to detrimental effects on mental health and well-being. Given the importance of social touch, there is growing interest in exploring the potential of artificial intelligence (AI) to supplement or even replicate human touch experiences. We are living in the AI era, and machines seem able to substitute humans in many fields of life, but may they become “human-like” in all aspects? Can they have the empathy and interpersonal skills of humans, or can they touch each other like us? If we think of newborns and the caress they receive, can this contact be substituted by a robot? A critical feature is the integration of AI in robots to create robots that are able to learn and make more complex tasks than simple and repetitive ones. These kinds of robots could be used to learn social touch and how to apply it in different physiological and pathological contexts in both adults and newborns. Robot therapy in medical and welfare facilities is spreading in our society, and AI could be a positive revolution in all touch-deprived situations, but further studies will need to be conducted to integrate robot therapy into our societies.

The results discussed in this review highlight the multifaceted benefits of social touch across various physiological and pathological conditions. From promoting immune system development in preterm newborns to alleviating symptoms of mood disorders in adults, social touch exerts positive effects on physical and mental health. Furthermore, the importance of touch emerges early in human development, with evidence suggesting that tactile stimulation in utero influences fetal responsiveness and development.

In recent years, AI has become increasingly integrated into various aspects of healthcare, offering opportunities for improved diagnosis, treatment, and personalized medicine. Deep-learning techniques have shown promise in medical imaging analysis and disease classification. Moreover, the integration of AI into robotic systems has led to advancements in prosthetic devices and robotic-assisted therapies. These technologies aim to enhance sensory feedback and improve motor control in individuals with limb loss or neurological disorders.

Robots integrated with AI offer the potential to simulate and even enhance human touch experiences. Developments in artificial skin and tactile sensors enable robots to detect and respond to touch stimuli with precision and sensitivity comparable to human touch. For example, the BioTac^®^ sensor, inspired by human fingertip anatomy, enables prosthetic hands to perceive force, vibration, and temperature, improving user capabilities and interaction with the environment. Similarly, AI-driven massage robots have been developed to provide therapeutic touch experiences, offering potential benefits for patients with various medical conditions.

However, while AI-driven technologies hold promises for supplementing human touch experiences, they also raise ethical and practical considerations. The development of robots that mimic human touch raises questions about the authenticity and emotional significance of tactile interactions. Behind the emotional aspects, there are critical factors that should be considered in the creation of a real human hand that can mimic a real touch, e.g., (1) the experience of different tissue characteristics (e.g., soft or wrinkled); (2) the sensation of hot or cold based on the temperature of touched objects, therefore, the reflex to immediately remove the hand; (3) the reflexes of human muscle; (4) the force of human grip based on the different characteristics of touched objects; or (5) the speed and pressure to use for a delicate or deep massage. In this context, the social robots are critical.

Moreover, concerns about privacy, data security, and equitable access to AI-driven healthcare technologies need to be addressed to ensure responsible and inclusive implementation.

Importantly, more studies are needed in this field to increase the possibilities of the use of robots in many fields requiring social touch as well as to investigate the social, psychological, and physical effects on individuals.

In conclusion, the integration of AI into touch-related technologies offers exciting possibilities for improving healthcare outcomes and enhancing human well-being. However, careful consideration of the ethical, social, and psychological implications is essential to ensure that these technologies are developed and deployed responsibly. Collaborative efforts between scientists, clinicians, policymakers, and ethicists are needed to navigate the complex landscape of AI-driven touch technologies and to maximize their potential benefits while minimizing potential risks.
